# Early and mid term mortality after coronary artery bypass grafting in women depends on the surgical protocol: retrospective analysis of 3441 on- and off-pump coronary artery bypass grafting procedures

**DOI:** 10.1186/1749-8090-5-90

**Published:** 2010-10-25

**Authors:** Sandra Eifert, Eckehard Kilian, Andres Beiras-Fernandez, Gerd Juchem, Bruno Reichart, Peter Lamm

**Affiliations:** 1 Department of Cardiac Surgery, Ludwig Maximilians University Munich; Munich, Germany

## Abstract

**Background:**

Since 2002 MI and stroke, not cancer, are leading causes of death in women. We studied 30-days and 1 year mortality of 3441 patients undergoing coronary artery bypass grafting (CABG) operations in our institution performed either conventionally or off pump (OPCAB). Our objective was to investigate the gender-related mortality in both groups.

**Patients and Methods:**

Between 2004 and 2008, 3441 patients (733 women, 2708 men) underwent CABG. 252 women and 854 men were operated using OPCAB, 481 women and 1854 men using extracorporeal circulation (ECC). Medical data was prospectively entered and retrospectively reviewed. 30-days and one year mortality rates were analyzed with Kaplan-Meier estimates and Cox proportional hazards models. Linear and logistic regression models were used to test gender differences.

**Results:**

a) 30-day mortality using ECC: 5.2% in women vs. 2.5% in men (p = 0.001). One year ECC mortality: 8.7% in women vs. 4.8% in men (p = 0.0008). b) OPCAB: 30-days and 1 year mortality in women measured 1.7%. Mortality in men was 2.1% after 30 days and 3.7% after one year c) gender specific mortality: 30 days mortality in women was 1.7% using OPCAB and 5.2% using ECC (p = 0.002), one year mortality in women was 1.7% using OPCAB vs. 8.7% using ECC (p = 0.0004). In men, 30-days mortality in OPCAB was 2.1%, one year mortality was 3.7%; using ECC early and late mortality was 2.5% and 4.8%.

**Conclusions:**

Female gender is a strong independent predictor and risk factor of increased early and midterm postoperative mortality rates when ECC is used. OPCAB significantly reduces early and midterm postoperative mortality in women and may therefore be proposed as the preferred revascularization technique in female patients.

## Background

Every year, 215,000 women die of cardiovascular diseases and approximately 30,000 women die of MI in Germany. Coronary artery bypass grafting (CABG) is one of the most frequent surgical procedures in the western world, among them approximately one third in women and between 6 to 10 per cent are operated off pump. Operative mortality in coronary surgery in women is much higher than compared to men. Several studies support these findings without explaining the causal reasons [1-3] Women, who have to undergo CABG, show a different risk profile than men and are treated less frequently pharmacologically in this regard. They suffer more frequently from diabetes, hyperlipidemia and arterial hypertension than men. Until menopause women are prevented from coronary artery disease (CAD) through estrogens, which have-among other facilities - a positive effect on lipid metabolism and cholesterol. With the hormone depletion during and after menopause this protection is weakening and that may be the reason, why CAD is occurring in women more frequently from the age of 60 years on [2].

The CABG operative mortality of women between 2004 and 2008 at our institution was 5.2% and thus, almost twice as high as in men (2.5%). Our aim was to investigate the mortality rate after CABG depending on the surgical protocol. Mortalities of men and women undergoing CABG under ECC or in OPCAB technique were compared. The present study was designed to observe 30 days and one year mortalities of patients undergoing CABG operation conventionally or in off pump technique. A reported exception is a previously published paper by Shroyer et al., showing that OPCAB mortality is not superior in comparison to conventional CABG. A major limitation of this paper was, that more than 99% of population was male [3]. Further goal of our study was to determine the gender-related mortality observed after CABG under ECC and compared to the gender-related mortality obtained after OPCAB.

## Methods

At the Cardiac Surgery Department of the Ludwig Maximilians University Munich 3441 patients (733 women, 2708 men) underwent CABG between January 2004 and July 2008 and were included in our study. Among these 3441 patients, 1006 patients (252 women and 854 men) were operated in off pump technique and 2335 (481 women and 1854 men) under extracorporeal circulation. Among the ECC cases, 10 were converted from OPCAB to conventional procedure due to ECG changes or intolerable hemodynamic changes. Excluded were emergency and redo cases as well as patients with valvular disease. Patient's medical data, prospectively entered and retrospectively reviewed, included demographic data as well as risk factors such as preexisting comorbidities, perioperative status, operative strategy, and clinical outcomes. Data was managed by local cardiovascular database "Kardiosoft". Each patient underwent a single surgical session consisting of OPCAB or CABG under ECC, at the discretion of the attending surgeon. This is a single center, retrospective study.

### Follow-up

Follow-up information of all patients dismissed from the hospital was obtained by an experienced coworker based on the follow-up letter every 6 months after the initial procedure for a duration of maximally 5 years. Information regarding vital status was sought.

### Statistical Analysis

Statistical data analysis was carried out by means of SPSS (Version 15.0, SPSS Inc., Chicago, IL, USA). Continuous data was summarized as mean ± standard deviation; discrete data were summarized as frequencies and group percentages. Linear and logistic regression models were used to test gender differences (test for interaction).

P values of ≤ 0.05 were considered significant. Furthermore, 30-day and one year mortality rates were analyzed with Kaplan-Meier estimates and Cox proportional hazards models. The 2 end points of interest were procedure related 30-day and one year mortalities between men and women operated in OPCAB technique and under ECC. Gender specific mortality was also obtained.

## Results and Discussion

### Pre and intraoperative Characteristics

Patient's baseline characteristics are summarized in Table 1. Table 2 and Table 3 provide preoperative and intraoperative data. BMI, Hyperlipidemia, Diabetes and AF rates were higher among women. Men smoked significantly more independent on surgical protocol (p = 0.000001). In women operated on-pump two vessel disease at a higher ejection fraction was leading, whereas three vessel disease was predominant among men operated under ECC conditions (p = 0.000001). Status post myocardial infarction had a higher incidence among male patients in comparison to women (p = 0.09). Men received more bypass grafts, specifically more arterial bypasses (n.s.).

**Table 1:** Patient's demographic data

	CABG on ECC		OPCAB	
	Men	Women	Men	Women
Number of Patients	1807	481	836	252
Age [Mean ± SD]	60.9 ± 7.4	65.5 ± 10.1	58.2 ± 8.4	66.2 ± 6.9
Ejection Fraction (%)	54.0 ± 9.5	58.9 ± 14.5	58.0 ± 2.7	64.3 ± 8.5
BMI [Mean ± SD]	27.4 ± 3.6	28.1 ± 4.5	24.9 ± 2.1	26.4 ± 3.2
Hypertension,%	78,5	63,7	79,1	59,7
Hyperlipidemia,%	75,2	78,5	78,8	79,3
Smoking ever,%	78,3	44,3	72,7	41,6
Diabetes mellitus,%	11,3	15	14,5	13,5

All p values are not significant among groups except for smoking ever: p = 0.000001 among sexes.

**Table 2:** Preoperative data

	CABG on ECC		OPCAB	
	Men	Women	Men	Women
NYHA Class	I: 11%	I: 3%	I: 9%	I: 2%
	II: 46,2%	II: 51%	II: 47,2%	II: 53,5%
	III: 53,8%	III: 41%	III: 54,8%	III: 43,5%
	IV: 0%	IV: 5%	IV: 2%	IV: 3%
Number of diseased coronary arteries	1: 0%	1: 27%	1: 2,2%	1: 25%
	2:30,8%	2:40%	2:42,8%	2:44,3%
	3: 69,2%	3: 33%	3: 55%	3: 30,5%
Left main stem disease	23%	18%	18,2%	16,1%
Former Myocardial Infarction	34,9%	24,8%	33,7%	25,1%
Former PTCA and Stenting	6,2%	6,4%	7,7%	5,1%
Atrial Fibrillation	22,7%	34,5%	20,3%	35,6%
Previous Stroke	4,4%	3,6%	3,8%	2,8%
Renal Failure	5,4%	4,6%	4,8%	3,8%

All p values are not significant among groups except for three vessel disease: p = 0.000001. For former myocardial infarction the p value measured 0.09 and thus showed a trend.

**Table 3:** Intraoperative data

	CABG on ECC		OPCAB	
	Men	Women	Men	Women
Time of Operation [minutes, Mean ± SD]	239,6 ± 102,5	222,7 ± 78,7	202,6 ± 55,7	192,6 ± 49,4
Cardiopulmonary Bypass Time [minutes, Mean ± SD]	130,3 ± 66,8	109,3 ± 40,1	-	-
Aortic Cross Clamp Time [minutes, Mean ± SD]	72,5 ± 30	65,6 ± 24,4	-	-
Time of Reperfusion [minutes, Mean ± SD]	40,8 ± 26,5	36,3 ± 19,8	-	-
Number of established bypasses [Mean ± SD]	2,43 ± 1,08	2,08 ± 1,17	2,28 ± 1,24	1,96 ± 1,74
Number of arterial bypass grafts [Mean ± SD]	1,26 ± 0,82	1,07 ± 0,54	1,18 ± 0,73	1,11 ± 0,32
Number of venous bypass grafts [Mean ± SD]	1,77 ± 1,03	1,02 ± 0,94	1,07 ± 1,53	1,12 ± 1,14
LITA to LAD	89%	87%	94%	92%
Total RA	21%	15%	2%	0%

All p values are not significant among groups.

### Postoperative Results

**Figure 1:**
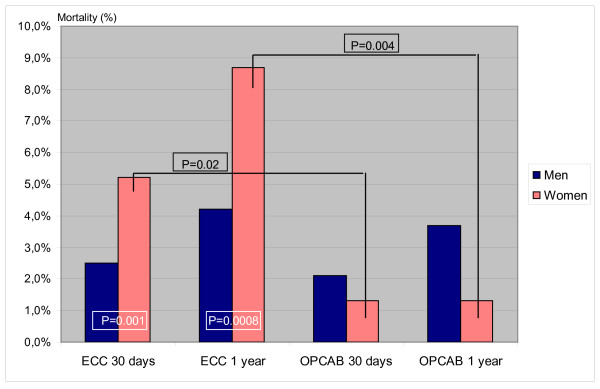
**Survival rates depending on operative technique under gender specific perspective**.

The postoperative results are summarized in Table 4 and Figure 1. Table 4 is reporting about postoperative complications whereas Figure 1 is documenting the survival rates

**Table 4:** Postoperative results

	CABG on ECC		OPCAB	
	Men	Women	Men	Women
Postoperative Resuscitation	3%	2%	1,1%	1%
Postoperative Myocardial Infarction	1%	1,5%	0,5%	0,5%
Neurological Disorders	6%	4%	1,5%	1%
Postoperative Atrial Fibrillation	25%	33%	23%	35%
Postoperative Acute Renal Failure	5%	2%	3,5%	2%
Wound Infection	2%	1,4%	1,8%	1,5%
Intraaortic Balloon Pump postoperatively	7%	3%	2,1%	1%
Rethoracotomy	8%	9%	4%	4%

All p values are not significant among groups.

### Procedure related mortality after 30 days and one year

The 30-day mortality using ECC measured 5.2% in women vs. 2.5% in men (p = 0.001). One year mortality showed a result of 8.7% in women vs. 4.8% in men (p = 0.0008). Using OPCAB technique, 30-day and 1 year mortality in women measured 1.7%. Mortality in men was 2.1% after 30 days and 3.7% after one year (not significant).

### Gender specific mortality

Thirty day mortality in women was 1.7% using OPCAB and 5.2% using ECC (p = 0.002), one year mortality in women was 1.7% using OPCAB vs. 8.7% using ECC (p = 0.0004). In men, 30-day mortality in OPCAB was 2.1% versus 2.5% under ECC. One year mortality derived from OPCAB technique measured 3.7%. Under ECC one year mortality was 4.8%, and thus among men not statistically significant. Women operated in OPCAB technique show the lowest operative mortality after 30 days and one year (1.7%). In addition, in our cohort men do not seem to benefit from OPCAB surgery (Figure 1).

## Discussion

Women undergoing CABG under ECC conditions present a higher mortality rate than men (average: 3.3% in men and 7.1% in women), as confirmed by numerous reports on gender differences in CABG procedures in the medical literature [4-10]. Although advances in myocardial preservation and ECC have allowed cardiac surgeons to perform conventional CABG, and other procedures with ECC safely and effectively, this gender-related difference in mortality remains constant, not only in CABG, but also in other cardiac surgical procedures under ECC, including congenital malformations in children.

However, the outcome after CABG under ECC in women as well as in men has improved over the last decades. The reasons for improvement noted in both genders are, in our opinion, most likely multi-factorial. Possible determinants of the current reduced mortality include newer technologies, improved surgeon's performance, and better education as well as more effective anti-aggregation treatments. However, risk profile and absolute adverse event rates in women are higher than in men, and this has not changed over the recent years as demonstrated by Puskas et al. in 42,477 consecutive patients. Furthermore, he reported that female patients were generally sicker and older than male patients at the time of operation [5,11]. Female gender was a strong, independent predictor of negative outcomes after CABG under ECC. We have observed a similar trend of outcome during CABG surgery under ECC, although risk factors were not that predominantly higher among women (BMI, Hyperlipidemia, Diabetes and AF rates were higher. In women operated on-pump two vessel disease at a higher ejection fraction was leading, whereas three vessel disease was predominant among men operated under ECC conditions. Men received more bypass grafts, specifically more arterial bypasses. This large contemporary data set confirms the historic gender disparity in clinical outcomes reported after CABG. Fu et al. [10] described a similar trend of early mortality as we did see in favor to women.

The higher mortality of women undergoing CABG can be discussed from different perspectives. According to symptomatic, the MONICA study revealed that roughly 80% of men with an acute MI suffer from angina, which in 50-60% radiates in the left arm [12]. Women report more frequently of oppression/constriction than men, but less of crucial deteriorating pain. Furthermore, women show frequently "atypical" symptoms such as nausea, vomiting and back pain. Regarding the co-morbidity, more women suffer from heart failure and atrial fibrillation [2]. The body surface area in women is smaller; the average hemoglobin level is lesser [13]. Regarding to the treatment, women visit doctors at a later time point, receive less drugs and undergo surgery more often as emergency patient. It has been also reported that women receive less bypass grafts during CABG, especially less arterial bypasses, due to their smaller body area. Perioperatively, the catecholamine dosage is higher compared to men. Respirator therapy has to be applied longer after CABG in comparison to men. Consecutively, women have a longer intensive care stay and therefore, a higher risk of pulmonary infection [14].

In conclusion, female gender seems to be a significant risk factor in many multivariate analyses. Therefore, in all important scoring systems for stratification of preoperative CABG risk, female gender has been defined a separate risk factor [15,16].

The discussion of CABG outcomes depending on the type of procedure remains controversial. As previously described by Shroyer et al., composite outcome of approximately 1000 patients operated on pump and OPCAB after 30 days was 5.6 vs. 7.0% respectively) (n.s.) and after one year measured 9.9 vs. 7.4%, respectively (p = 0.04). In this specific study, basically all investigated patients were men [3].

In our cohort, the 30-day and one year mortality using ECC was significantly higher among women, while there were no significant differences using OPCAB technique. Looking at mortality rates under a gender-specific perspective in our patient's population, OPCAB is most and specifically favourable for women. OPCAB has been performed for many years. Its use is increasing in frequency, and it remains an open question why OPCAB is associated with better outcomes than on-pump CABG surgery.

Our results reflect a drastically lower mortality in woman after OPCAB. The mortality rates in men and women from the retrospective study coming from a single center suggest the recommendation of exclusive OPCAB use in women undergoing CABG. Larger prospective randomized studies in the near future should be carried out to support our preliminary results.

## Conclusions

Female gender is a strong independent predictor and risk factor of increased postoperative mortality rates when ECC is used. OPCAB significantly reduces early and midterm postoperative mortality in women and may therefore be proposed as the preferred revascularization technique among female patients.

## Competing interests

The authors declare that they have no competing interests. Institutional review board approval was received before investigations have been started.

## Authors' contributions

B.24.1 SE, EK, GJ, BR, PL have made substantial contributions to conception and design, or acquisition of data, surgical procedure and interpretation of data; SE, ABF and PL have been involved in drafting the manuscript or revising it critically for important intellectual content; and all authors have read and given final approval of the version to be published.
